# LncRNA FLG-AS1 inhibits esophageal squamous cell carcinoma by regulating the miR-23a-3p/HOXD10 axis

**DOI:** 10.1186/s41065-025-00461-0

**Published:** 2025-06-03

**Authors:** Zhigao Zhang, Fucheng Zhang, Chuan Xue, Xiaoling Song, Yaojun Wang

**Affiliations:** 1 Department of Gastroenterology, Sunshine Union Hospital, No. 9000, Yingqian Street, Gaoxin District, Weifang City, 261000 Shandong Province China; 2 Department of Gastroenterology I, Shandong Second Provincial General Hospital, Jinan City, 250021 Shandong Province China

**Keywords:** Esophageal squamous cell carcinoma, Proliferation, Migration, Cisplatin resistance

## Abstract

**Background:**

Esophageal cancer (EC) is the ninth most common cancer worldwide that kills about 300,000 people each year. Esophageal squamous cell carcinoma (ESCC) is the main type of EC. Long non-coding RNAs (lncRNAs) have been proven to be severely dysregulated in EC, but the functions of more lncRNAs still need to be explored.

**Methods:**

To explore the new molecular mechanism of ESCC development, the online biology databases (GEO, lncRNASNP2, Starbase, TargetScan) were employed to investigate the novel pathways implicated. To assess the expression levels of FLG-AS1, miR-23a-3p, and associated genes, we utilized RT-qPCR. The expression of HOXD10 was evaluated through western blotting analysis. To elucidate the regulatory interactions among FLG-AS1, miR-23a-3p, and HOXD10, a combination of dual luciferase assays, silencing techniques, and overexpression studies were conducted. The migratory and invasive capabilities of the cells were examined using a transwell apparatus. Cell viability was measured employing the CCK-8 assay, while apoptosis was detected through Annexin V/PI double staining methodology. Concentrations of glucose and lactic acid were determined utilizing appropriate biochemical kits.

**Results:**

FLG-AS1 and HOXD10 exhibited low expression levels in ESCC cells, whereas miR-23a-3p was found to be highly expressed. FLG-AS1 was observed to reduce the free level of miR-23a-3p by directly binding to it, and in turn, miR-23a-3p inhibited the expression of HOXD10 by targeting its mRNA. The overexpression of FLG-AS1 and HOXD10 resulted in the attenuation of anaerobic glycolysis, as well as a decrease in the migratory and invasive capabilities of ESCC cells, effectively reversing their resistance to cisplatin. Conversely, the upregulation of miR-23a-3p yielded opposing effects. Furthermore, ESCC patients exhibiting elevated levels of FLG-AS1 and HOXD10, alongside reduced expression of miR-23a-3p, demonstrated a significantly higher 5-year survival rate post-surgery.

**Conclusion:**

FLG-AS1 effectively inhibits the progression of ESCC and counters cisplatin resistance through the modulation of the miR-23a-3p/HOXD10 axis. This is a new mechanism affecting ESCC and will provide new ideas for the targeted therapy of ESCC.

**Supplementary Information:**

The online version contains supplementary material available at 10.1186/s41065-025-00461-0.

## Introduction

Esophageal cancer (EC) represents a prevalent malignancy within the digestive tract. Early manifestations often include a sensation of foreign body obstruction during the ingestion of food and retrosternal pain. In the intermediate to advanced stages, patients typically experience progressive dysphagia, while those with metastasis may also suffer from pain or discomfort in the affected organs [1, 2]. EC is principally classified into two histological types: esophageal squamous cell carcinoma (ESCC) and esophageal adenocarcinoma, with ESCC constituting over 90% of cases. According to estimates for 2022, there were approximately 511,054 new diagnoses of EC, resulting in 445,391 fatalities worldwide [3]. As the population continues to grow and age, the burden of age-related EC is anticipated to rise significantly. The primary treatment modality for EC is surgical resection, which may involve either thoracotomy or laparoscopic approaches to excise the tumor. In cases of early-stage EC, radical surgical intervention can lead to a cure; however, a significant number of patients are diagnosed at an advanced stage, often with distant metastases, rendering surgical options largely ineffective. For these individuals, conservative treatment strategies such as chemoradiotherapy or targeted therapy become the only viable alternatives [4]. Consequently, a critical correlation exists between prognosis and the timeliness of EC detection. Early identification and prompt intervention can elevate the 5-year survival rate for EC to over 90% [5].

The investigation into the molecular mechanisms of EC is vital for enhancing the efficacy of targeted therapies. In recent years, the competitive endogenous RNA (ceRNA) network has garnered significant attention within the cancer research community. MicroRNAs (miRNAs), which are evolutionarily conserved, regulate the activity and stability of target genes by recognizing the miRNA response elements (MRE) within the 3’UTR region of target mRNAs, subsequently facilitating the assembly of gene silencing complexes known as the RNA-induced silencing complex (RISC). Intracellular ceRNAs, such as long non-coding RNAs (lncRNAs) and circular RNAs (circRNAs), also harbor MREs. When ceRNAs and mRNAs possess identical MREs, they establish a competitive relationship for the same miRNA, allowing ceRNAs to sequester miRNAs through the MRE bridge, thus indirectly modulating mRNA expression and influencing cellular functions [6–8]. Recent studies have elucidated the critical roles of lncRNA-miRNA-mRNA interactions and their dysregulation in the pathophysiology of EC. Notably, LINC00657 has been shown to be aberrantly expressed in EC cells, functioning as a sponge for miR-26a-5p. It was observed that CKS2 serves as a direct target for miR-26a-5p, and when LINC00657 is knocked out, there is an upregulation of miR-26a-5p, resulting in the inhibition of CKS2 expression and a consequent suppression of EC cell proliferation [9]. Furthermore, lncRNA TTN-AS1, identified as an oncogene in ESCC, promotes the expression of the transcription factor Snail1 through competitive binding with miR-133b, thereby facilitating the epithelial-mesenchymal transition (EMT) process; additionally, it can enhance ESCC invasion by sponging miR-133b to induce the expression of FSCN1 [10]. Moreover, other interactions among lncRNA H19/miR-222/CBX2, H19/miR-330/PIK3R4, and lncRNA KCNQ1OT1/miR-374a/VEGFA have been identified, all of which play potentially significant roles in the metastasis of ESCC [11].

The intricate mechanisms underlying other ceRNA networks in ESCC remain largely uncharted. Notably, LncRNA FLG-AS1 has been documented to inhibit cervical cancer cell functions through the down-regulation of miR-147b [12]. Thus, we posited that FLG-AS1 may function as a ceRNA, modulating the miRNA/mRNA axis to influence the progression of ESCC. By employing bioinformatics techniques, we constructed a comprehensive lncRNA-miRNA-mRNA interaction network, which enabled us to identify the miR-23a-3p/HOXD10 axis as a significant downstream target of FLG-AS1 in our study. Furthermore, we meticulously analyzed the implications of this regulatory network on ESCC metastasis and cisplatin resistance through carefully designed experiments, while also assessing its predictive value concerning surgical outcomes for ESCC patients.

## Materials and methods

### Target prediction

The publicly accessible datasets GSE130078, GSE111011, and GSE137867 were sourced from the Gene Expression Omnibus (GEO) database (https://www.ncbi.nlm.nih.gov/geo/). The expression data in these datasets were analyzed for differential expression by means of the GEO2R analysis tool in the GEO database. The GEO2R function is based on the package of R language. To predict downstream miRNAs of lncRNAs, we utilized the lncRNASNP2 database (https://guolab.wchscu.cn/lncRNASNP#!/) and the Starbase database (https://rnasysu.com/encori/index.php). The upstream miRNAs of genes were predicted using the TargetScan database (https://www.targetscan.org/vert_80/). The identified lncRNAs, miRNAs, and genes were integrated into a network diagram utilizing Cytoscape 3.7.1 software, whereby key pathways associated with ESCC were identified through the calculation of node degree value.

### Cells and treatment

Human esophageal epithelial cells (hEECs) and human esophageal squamous cell lines, specifically KYSE-30 and KYSE-410, were sourced from Pricella Biotechnology Company (China) and cultivated utilizing the company’s proprietary complete culture medium (CM-H031, CM-0577, and CM-0586). The cisplatin-resistant cell line KYSE30/DDP was obtained from the Cell Bank of the Type Culture Collection of the Chinese Academy of Sciences and maintained in high-glucose DMEM (Sigma-Aldrich, USA) supplemented with 10% fetal bovine serum (Gibco, USA) and 1% penicillin/streptomycin (Solarbio, China). All cell lines were incubated at 37 °C in an atmosphere containing 5% CO_2_.

Parental KYSE-30 cells were subjected to treatment with increasing concentrations of cisplatin (MedChemExpress, America), specifically at doses of 2, 4, 6, 8, 10, and 12 µg/mL. Meanwhile, KYSE30/DDP cells were treated with higher concentrations, namely 10, 20, 30, 40, 50, and 60 µg/mL of cisplatin, for a duration of 24 h. This experimental design aimed to evaluate the semi-inhibitory concentration of cisplatin in both cell lines.

### RT-qPCR

Total RNA was extracted utilizing the Trizol reagent from Beyotime (China). Quantitative PCR was conducted employing the BeyoFast™ SYBR Green One-Step RT-qPCR Kit provided by Beyotime. The internal reference gene for lncRNA and mRNA was GAPDH, while miRNA was normalized using U6 [13–15]. The primer sequences are detailed in Supplementary Table 1. The relative expression levels of lncRNA, miRNA, and genes were calculated using the 2^−ΔΔCt^ method.

### Dual luciferase assay

DNA fragments harboring wild-type or mutant FLG-AS1/HOXD10 3’UTR sequences were meticulously cloned into the psiCHECK-2 vector. KYSE-30 cells were seeded into a 48-well plate at a density of 5 × 10^4^ cells per well, allowing for adherence 24 h prior to transfection. MiR-23a-3p mimics or inhibitors (obtained from MedChemExpress) were co-transfected alongside the luciferase vectors using Lipofectamine 2000 (Beyotime). Prior to transfection, the growth medium was replaced with 225 µL of Opti-MEM medium (MeilunBio, China) for each well. The transfection mixture comprised 25 µL and included luciferase vectors (2.5 µg) or miR-23a-3p mimics/inhibitors (10 nM), supplemented with 6 µL of Lipofectamine 2000 and the corresponding volume of Opti-MEM medium. Following a 15-minute incubation period for the transfection mixture, it was added to the cells in each well. After a duration of 48 h, luciferase activity within the cells was assessed using the Luciferase Reporter Assay Kit (Vazyme, China). The activity of firefly luciferase was subsequently normalized against that of sea kidney luciferase.

### Western blotting

The cells were incubated on ice with RIPA lysis buffer (Applygen, China), supplemented with a protease inhibitor, for a duration of 30 min. Following centrifugation at 4 °C (12,000 rpm for 20 min), the resultant supernatant constituted the total cell protein extract. A total of 40 µg of this protein was then subjected to separation via a 10% polyacrylamide gel, and subsequently transferred to a polyvinylidene fluoride (PVDF) membrane. After a 2-hour incubation at room temperature in a blocking solution containing 5% skim milk powder, the PVDF membrane was incubated overnight at 4 °C with primary antibodies, followed by a 1.5-hour incubation at room temperature with the appropriate secondary antibody. The PVDF membrane was then washed with Tris-Borate-Sodium Tween-20 (TBST) buffer and detected using an enhanced chemiluminescence (ECL) luminescent solution (Proteintech, China). The primary antibodies targeting HOXD10 (1:1000) and GAPDH (1:2000) were obtained from Santa Cruz Biotechnology (USA), while the mouse secondary antibody conjugated with horseradish peroxidase (1:10,000) was sourced from ZSGB-BIO (China).

### Transwell migration and invasion assay

The migration and invasion capacities of ESCC cells were assessed using 0.4 μm transwell chambers (Corning, America) [16, 17]. The upper chambers of the transwell, either devoid of or supplemented with matrigel, were utilized for measuring migration and invasion, respectively. ESCC cells (6 × 10^5^) were seeded into the upper chamber in serum-free medium, while 1000 µL of serum-containing medium was introduced into the lower chamber. Following a 24-hour incubation period, the submembrane cells were fixed with 4% paraformaldehyde (Beyotime) for 10 min and subsequently stained with 0.5% crystal violet (Beyotime) for 15 min. Finally, the stained cells were counted under a microscope.

### Evaluation of anaerobic glycolysis in ESCC cells

Anaerobic glycolysis was assessed by investigating glucose utilization, lactate production, and the expression of the glucose transporter GLUT3 in ESCC cells. To measure glucose concentration and lactate levels in the cell culture medium, we employed the Glucose Assay Kit from Abcam and the Lactic Acid Content Assay Kit from Solarbio.

### Cell viability assay

The proliferation of ESCC cells was assessed using the Cell Counting Kit-8 (CCK-8) reagent (Solarbio). ESCC cells were seeded into 96-well plates at a density of 8 × 10^3^ cells per well and subjected to the indicated treatments. Following treatment, the existing medium was carefully aspirated and replaced with 100 µL of fresh medium supplemented with 10% CCK-8 reagent. The plates were then incubated at 37 °C for 2 h, after which the optical density (OD) of each well was measured at a wavelength of 450 nm using an enzyme labeling. Cell viability was calculated as a percentage based on the manufacturer’s protocol using the following formula: [(As - Ab) / (Ac - Ab)] × 100%. As, the OD value of the test group (cell + drug). Ab, the OD value of the blank group (cell-free). Ac, the OD value of control group (cell without drug).

### Apoptosis detection

The Annexin V-FITC Apoptosis Detection Kit (Beyotime) serves as an essential tool for assessing cell apoptosis. The procedure commences with the collection of treated cells, followed by centrifugation at 1000 g for 5 min, after which the supernatant is carefully discarded. Subsequently, 195 µL of Annexin V-FITC binding solution is added to the pellet, followed by the careful addition of 5 µL of Annexin V-FITC and 10 µL of propidium iodide (PI) staining solution, ensuring gentle mixing to achieve a homogeneous suspension. The cells are then incubated at room temperature, shielded from light, for a duration of 10 to 20 min. Finally, the apoptotic cells are quantified through flow cytometry, allowing for accurate determination of the apoptotic cell population.

### Clinical samples

Utilizing online statistical tools (https://select-statistics.co.uk/), the recommended minimum sample size was calculated to be 95. To account for potential sample attrition, 100 healthy volunteers and 123 patients diagnosed with ESCC were enrolled from *Sunshine Union Hospital*. Peripheral blood samples were obtained from all participants. Key demographic and clinical characteristics of the study cohort are presented in Table 1. Notably, none of the ESCC patients had undergone prior radiation therapy or chemotherapy before their surgical intervention. This study was conducted following the acquisition of informed consent from all subjects and with the approval of the Ethics Committee of *Sunshine Union Hospital*.

**Table 1:** The basic information of participants

Group	Number (*n*)	Age (years)	Gender (F/M)	Smoking (*n*)	Drinking (*n*)	BMI (kg/m^2^)	Family history (*n*)
Healthy volunteers	100	60.0 ± 6.4	45/55	49	43	23.0 ± 2.1	4
ESCC patients	123	59.3 ± 6.8	57/66	68	63	25.1 ± 2.4	7

Notes: ESCC, esophageal squamous cell carcinoma; F, female; M, male; BMI, body mass index

### Prognostic value assessment

In this study, we meticulously monitored the survival duration of ESCC patients who underwent surgical intervention over a five-year period. Based on the levels of FLG-AS1, miR-23a-3p, and HOXD10 in postoperative blood samples, patients were stratified into high-expression and low-expression cohorts determined by calculating the median values. Utilizing GraphPad software, we constructed survival curves to elucidate the prognostic significance of the FLG-AS1/miR-23a-3p/HOXD10 axis in predicting outcomes following ESCC surgery.

### Statistical analysis

The statistical analyses for this study were conducted using GraphPad Prism 9.0 software. Data normality was assessed via the Shapiro-Wilk test. Comparisons between two groups were performed using Student’s t-tests. For datasets involving three or more groups, one-way analysis of variance (ANOVA) was employed. Statistical significance was defined as a *p*-value of < 0.05. Effect size analysis for clinical samples was performed using SPSS software; a partial η2 value greater than 0.14 was interpreted as indicative of a large effect size.

## Results

### Bioinformatics screening of important pathways involved in ESCC

The GSE130078 and GSE111011 datasets, derived from high-throughput RNA sequencing and published in the GEO database, were utilized to identify differentially expressed lncRNAs in ESCC tissues compared to adjacent normal tissues. Notably, FLG-AS1, LINC02487, LINC02621, and LINC02538 emerged as common lncRNAs (as illustrated in Supplementary Fig. 1A). To predict their downstream miRNAs, the lncRNASNP2 and starbase databases were employed. However, LINC02621 and LINC02538 were excluded from further analyses due to the absence of identifiable downstream miRNAs.

In our analysis, we meticulously screened for differentially expressed genes (DEGs) within ESCC tumor tissues compared to adjacent normal tissues through the GSE130078 and GSE111011 datasets. Additionally, we investigated the DEGs present in ESCC tissues before and after radiotherapy, utilizing the GSE137867 dataset. Notably, we identified SPP1, HOXD10, HOXD11, and PIGR as common DEGs across these datasets (see Supplementary Fig. 1B). Subsequently, we explored the upstream miRNAs associated with these four genes using the TargetScan database.

**Fig. 1:**
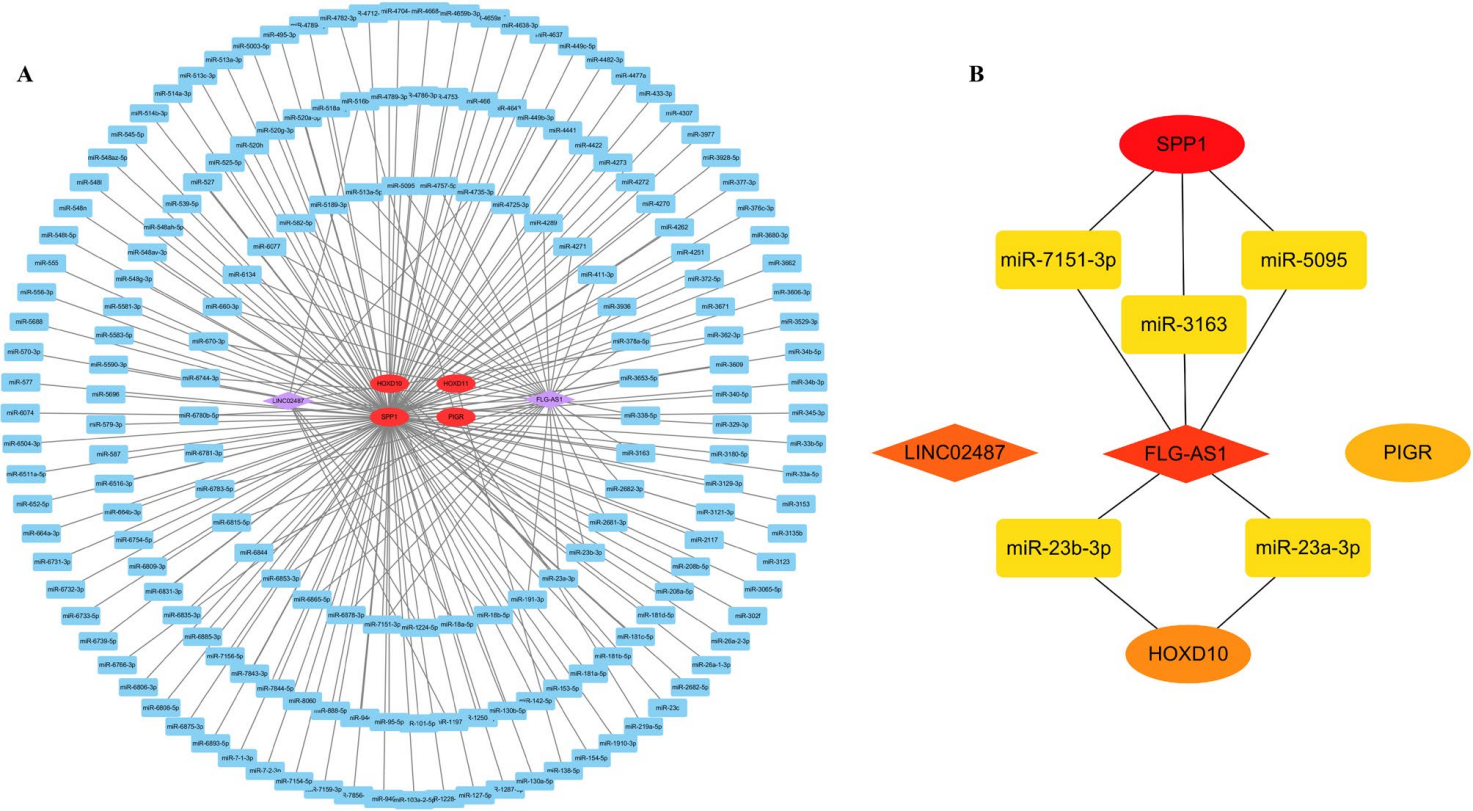
FLG-AS1/miR-23a-3p/HOXD10 was screened as an important pathway in ESCC. **(A)** The lncRNA-miRNA-mRNA network which is related to ESCC. **(B)** The top 10 molecules with high nodal degree values in the network diagram

Subsequently, we constructed a lncRNA-miRNA-mRNA network utilizing Cytoscape software (Fig. 1A), from which we identified the top 10 molecules based on their node-degree values (Fig. 1B). This analysis revealed two principal regulatory networks mediated by FLG-AS1. Given the extensive research on SPP1 within the context of ESCC, we opted to focus on HOXD10, a gene that has received comparatively limited attention. Notably, both miRNAs involved in the HOXD10 network have also been less explored in ESCC; thus, we selected one of these miRNAs for further mechanistic investigation.

### MiR-23a-3p mediated the promotion of HOXD10 expression by FLG-AS1

**Fig. 2:**
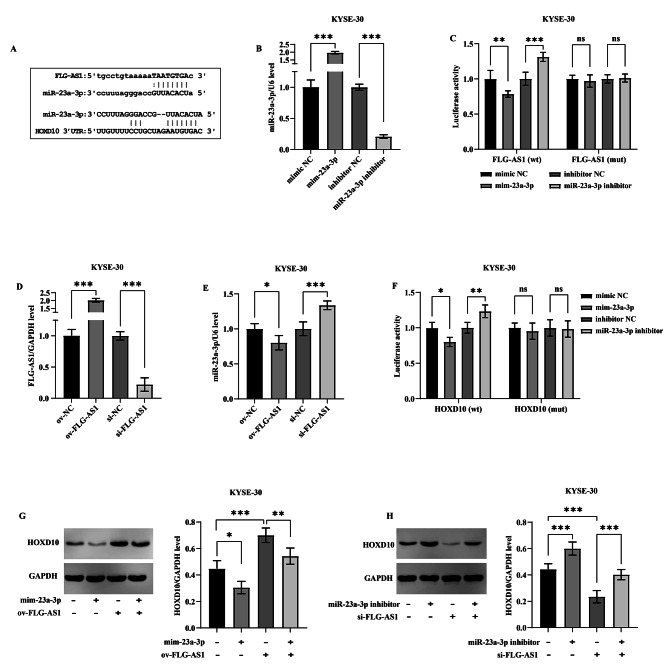
The regulation of miR-23a-3p and HOXD10 by FLG-AS1. (**A**) The target sequences of FLG-AS1 to miR-23a-3p and miR-23a-3p to HOXD10 mRNA. **(B)** The mimics and inhibitor of miR-23a-3p. **(C)** Dual luciferase assay verified the binding relationship between FLG-AS1 and miR-23a-3p. **(D)** The overexpression plasmid and small interfering RNA FLG-AS1. **(E)** Effect of overexpression or knockdown of FLG-AS1 on miR-23a-3p level. **(F)** Dual luciferase assay detected the interaction between miR-23a-3p and HOXD10 mRNA. **(G)** Effect of up-regulation of FLG-AS1 or miR-23a-3p on HOXD10 expression. **(H)** Effect of down-regulation of FLG-AS1 or miR-23a-3p on HOXD10 expression. *p < 0.05; **p < 0.01; ***p < 0.001; ns, no significant difference

Wild-type and mutant luciferase vectors of FLG-AS1 (wt/mut-FLG-AS1) and HOXD10 (wt/mut-HOXD10) were constructed based on the binding sequences of miR-23a-3p with the 3’ untranslated regions (UTRs) of FLG-AS1 and HOXD10 (Fig. 2A), aimed at elucidating their targeting relationships. Transfection with miR-23a-3p mimics (mim-23a-3p) resulted in a significant upregulation of miR-23a-3p cellular level, whereas its inhibitor effectively reduced its level (Fig. 2B). The dual luciferase assay validated the interaction between FLG-AS1 and miR-23a-3p, revealing that luciferase activity in cells transfected with wt-FLG-AS1 diminished upon treatment with miR-23a-3p mimics and increased following the application of its inhibitor. In stark contrast, the luciferase activity in cells harboring mut-FLG-AS1 remained unaltered by these treatments (Fig. 2C). The level of FLG-AS1 in the cells was markedly elevated following the transfection of an overexpression plasmid (ov-FLG-AS1), and could be effectively silenced by the corresponding small interfering RNA (si-FLG-AS1) (Fig. 2D). Notably, the overexpression of FLG-AS1 correlated with a downregulation of miR-23a-3p, while the silencing of FLG-AS1 led to an increase in miR-23a-3p level. These findings suggest that FLG-AS1 modulated the intracellular level of free miR-23a-3p by binding to it, thereby influencing its availability (Fig. 2E).

The dual luciferase assay revealed that the introduction of miR-23a-3p mimics resulted in a significant reduction in luciferase activity in cells transfected with wild-type HOXD10, while the application of its inhibitor led to an enhancement of luciferase activity. In contrast, no considerable effect was observed in cells transfected with the mutant HOXD10 (Fig. 2F). Furthermore, the expression level of HOXD10 was suppressed following the upregulation of miR-23a-3p, whereas downregulation of miR-23a-3p correspondingly increased HOXD10 expression (Figs. 2G and H). These findings suggest that miR-23a-3p exerts a negative regulatory influence on HOXD10 by directly targeting its mRNA.

Additionally, the expression of HOXD10 was found to be augmented by the overexpression of FLG-AS1, a phenomenon that was reversed upon co-transfection with miR-23a-3p mimics (Fig. 2G). Conversely, silencing FLG-AS1 led to a downregulation of HOXD10 expression, which could be restored by the inhibition of miR-23a-3p (Fig. 2H). Notably, the binding sequences of miR-23a-3p in both FLG-AS1 and HOXD10 mRNA were found to overlap (Fig. 2A), indicating that FLG-AS1 acts as a competing endogenous RNA, sequestering miR-23a-3p and thereby preventing its interaction with HOXD10 mRNA, ultimately promoting HOXD10 expression.

### FLG-AS1 inhibited the metastasis of ESCC cells by regulating the miR-23a-3p/HOXD10 axis

**Fig. 3:**
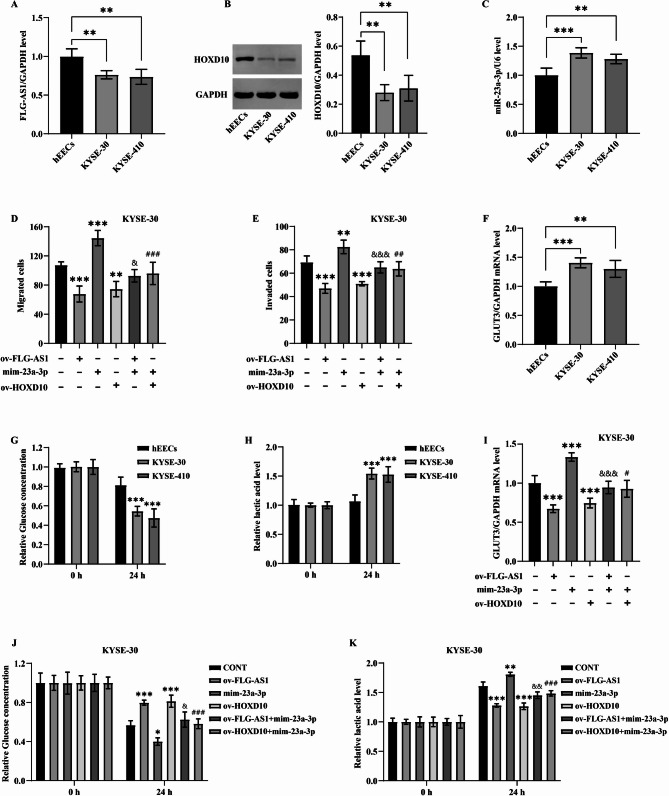
Inhibition of FLG-AS1 on ESCC cell metastasis by regulating the miR-23a-3p/HOXD10 axis. (**A-C**) FLG-AS1, HOXD10, and miR-23a-3p levels in hEECs and ESCC cells. **(D and E)** Roles of FLG-AS1, miR-23a-3p, or HOXD10 on the migratory and invasive abilities of ESCC cells. **(F)** The expression of GLUT3 in hEECs and ESCC cells. **(G)** Glucose concentration in the culture medium of hEECs and ESCC cells. **(H)** Lactic acid content in the culture medium of hEECs and ESCC cells. **(I-K)** Effect of overexpression of FLG-AS1, miR-23a-3p, or HOXD10 on GLUT3 expression, glucose utilization, and lactic acid production of ESCC cells. *p, ^#^*p*, and ^&^*p* < 0.05; **p, ^##^*p*, and ^&&^*p* < 0.01; ***p, ^###^*p*, and ^&&&^*p* < 0.001

Compared to esophageal epithelial cells, the expression levels of FLG-AS1 and HOXD10 were markedly diminished in ESCC cells (Fig. 3A and B), whereas the level of miR-23a-3p was notably elevated (Fig. 3C). Overexpression of FLG-AS1 and HOXD10 significantly curtailed cell migration, in stark contrast to the effects of miR-23a-3p, which facilitated the migration of KYSE-30 cells and countered the inhibitory effects of FLG-AS1 on ESCC cell motility. Co-transfection with a HOXD10 plasmid effectively mitigated the migratory enhancement induced by miR-23a-3p mimics (Fig. 3D). Similarly, the invasive capabilities of KYSE-30 cells were suppressed by the overexpression of FLG-AS1 and HOXD10, while they were augmented by the application of miR-23a-3p mimics. Furthermore, co-transfection with miR-23a-3p mimics partially abrogated the suppressive effects of FLG-AS1 on ESCC cell invasion, and co-transfection with the HOXD10 plasmid diminished the enhancing influence of the miR-23a-3p mimics (Fig. 3E).

Cancer cells often engage in anaerobic glycolysis even under aerobic conditions, leading to an enhanced consumption of glucose and the production of lactic acid, which contributes to an optimal microenvironment for their proliferation. In our study, we observed a pronounced overexpression of GLUT3 in ESCC cells compared to hEECs (Fig. 3F). Furthermore, ESCC cells demonstrated a significantly greater glucose uptake than normal cells over the same duration (Fig. 3G). Additionally, these cancer cells exhibited a heightened production of lactic acid relative to hEECs during the same time frame, correlating with their elevated glucose consumption (Fig. 3H).

The overexpression of FLG-AS1 and HOXD10 in KYSE-30 cells led to a significant reduction in GLUT3 expression and glucose uptake. In contrast, the introduction of miR-23a-3p mimics resulted in an enhancement of these processes. Notably, the upregulation of miR-23a-3p diminished the inhibitory effect of FLG-AS1 on GLUT3 expression and glucose uptake in KYSE-30 cells. Furthermore, the stimulatory effects of miR-23a-3p on these metabolic processes were effectively counteracted by the overexpression of HOXD10 (Fig. 3I-J). Additionally, lactic acid production in KYSE-30 cells was markedly increased upon transfection with miR-23a-3p mimics, while it was inhibited by the overexpression of FLG-AS1 and HOXD10. Importantly, the influence of FLG-AS1 and miR-23a-3p on lactic acid production was reversed by transfection with miR-23a-3p mimics or the overexpression of HOXD10, respectively (Fig. 3K). Collectively, these findings elucidate that the FLG-AS1/miR-23a-3p/HOXD10 signaling pathway impedes the migratory and invasive capabilities of ESCC cells by modulating their glycometabolic processes.

### The FLG-AS1/miR-23a-3p/HOXD10 axis in the cisplatin resistance

**Fig. 4:**
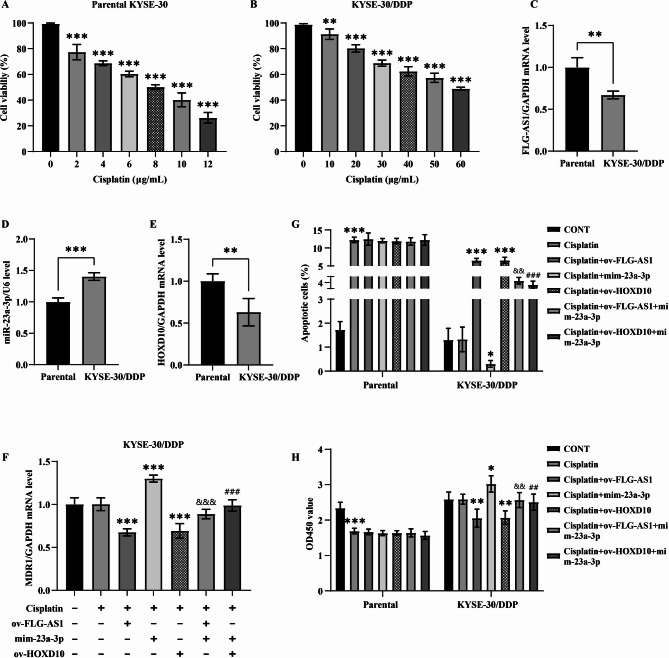
The FLG-AS1/miR-23a-3p/HOXD10 axis in the cisplatin resistance. **(A** and **B**) Effects of different concentrations of cisplatin on the viability of parental KYSE-30 and KYSE-30/DDP. **(C-E)** The expression of FLG-AS1, miR-23a-3p, and HOXD10 in parental KYSE-30 and KYSE-30/DDP cells. **(F-H)** Effects of up-regulation of the FLG-AS1/miR-23a-3p/HOXD10 axis on MDR1 expression, apoptosis, and cell viability of KYSE-30/DDP. *p < 0.05; **p, ^##^*p*, and ^&&^*p* < 0.01; ***p, ^###^*p*, and ^&&&^*p* < 0.001

Cisplatin, a widely utilized chemotherapeutic agent in cancer treatment, exhibited a significant inhibition of cell viability in parental KYSE-30 cells in a concentration-dependent manner. The half-maximal inhibitory concentration (IC50) for KYSE-30 cells was determined to be 8 µg/mL (refer to Fig. 4A). Conversely, while high concentrations of cisplatin also inhibited the activity of KYSE-30/DDP cells, a concentration of 60 µg/mL induced a semi-inhibitory effect (as shown in Fig. 4B). Notably, the IC50 of cisplatin for KYSE-30/DDP was found to be 7.5 times greater than that of the parental cells, thereby confirming the development of cisplatin resistance in this particular cell line.

In KYSE-30/DDP cells, the expression levels of FLG-AS1 and HOXD10 were notably reduced, whereas miR-23a-3p exhibited increased expression (Fig. 4C-E). The overexpression of FLG-AS1 and HOXD10 facilitated a corresponding rise in multidrug resistance protein 1 (MDR1) level in cisplatin-treated KYSE-30/DDP cells, an effect that was mitigated by the upregulation of miR-23a-3p. Notably, the mimics of miR-23a-3p effectively inhibited MDR1 expression, a suppression that could be rescued by the overexpression of HOXD10 (Fig. 4F). Exposure to 8 µg/mL cisplatin induced apoptosis in parental KYSE-30 cells; however, KYSE-30/DDP cells displayed resistance to this apoptosis. Remarkably, the overexpression of FLG-AS1 and HOXD10 reversed the cisplatin resistance in KYSE-30/DDP cells and subsequently promoted apoptotic activity. In contrast, the upregulation of miR-23a-3p enhanced the anti-apoptotic potential of KYSE-30/DDP, a phenomenon that could be reversed through the co-transfection with a HOXD10 plasmid. Furthermore, the elevation of miR-23a-3p was observed to hinder the apoptotic induction in KYSE-30/DDP cells mediated by FLG-AS1 (Fig. 4G). Similarly, 8 µg/mL cisplatin did not significantly hinder the proliferation of KYSE-30/DDP cells; indeed, these cisplatin-resistant ESCC cells demonstrated a superior growth capacity compared to their parental counterparts. Following the upregulation of FLG-AS1 and HOXD10 in KYSE-30/DDP, the growth of these cells was significantly inhibited by treatment with 8 µg/mL cisplatin, an effect that was diminished with the elevated levels of miR-23a-3p (Fig. 4H). Collectively, these results underscore the facilitative role of FLG-AS1 in enhancing the sensitivity of KYSE-30/DDP cells to cisplatin, operating through the modulation of the miR-23a-3p/HOXD10 axis.

### Clinical value of the FLG-AS1/miR-23a-3p/HOXD10 axis in ESCC

**Fig. 5:**
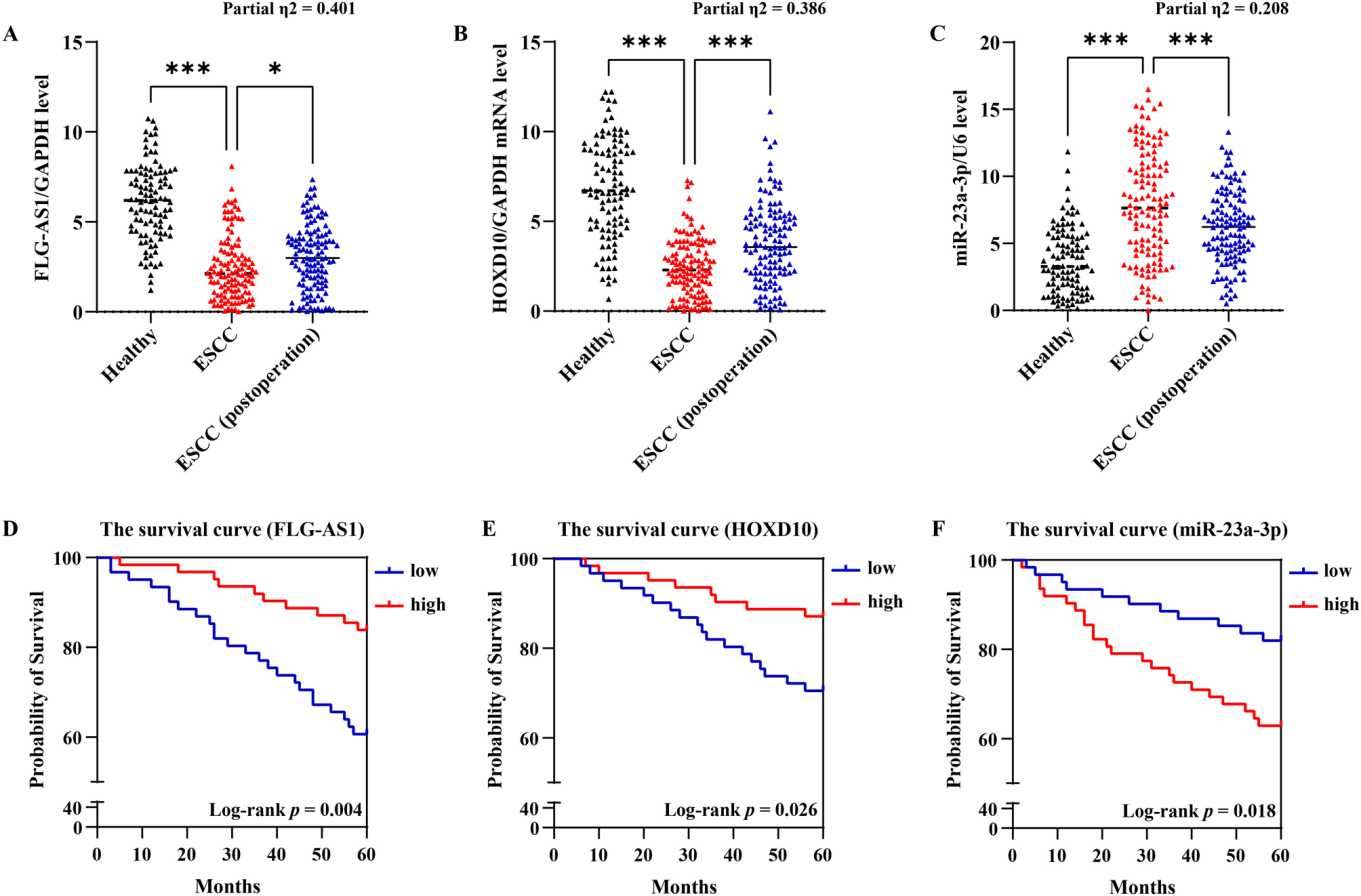
The prognostic value of the FLG-AS1/miR-23a-3p/HOXD10 axis. **(A-C)** The expression of FLG-AS1, miR-23a-3p, and HOXD10 in the peripheral blood samples of healthy volunteers and ESCC patients. **(D-F)** The predictive value of FLG-AS1, miR-23a-3p, or HOXD10 level in postoperative survival time of ESCC patients. *p < 0.05, ***p < 0.001

The results of RT-qPCR conducted on clinical samples indicated a notable deficiency of FLG-AS1 and HOXD10 in the peripheral blood of patients with ESCC. Remarkably, post-surgical interventions led to a significant elevation in the expression levels of these markers (Fig. 5A and B). Conversely, miR-23a-3p exhibited heightened expression in ESCC patients, with surgical treatment correlating with a reduction in its levels (Fig. 5C). Based on the post-operative expression profiles of FLG-AS1, miR-23a-3p, and HOXD10, the ESCC patients were stratified into low-expression and high-expression groups using the median as a cutoff. Follow-up analyses revealed that patients exhibiting high levels of FLG-AS1 and HOXD10 demonstrated superior 5-year survival rates compared to those with low expression (Fig. 5D and E). In contrast, lower expression levels of miR-23a-3p were associated with an enhanced 5-year survival rate (Fig. 5F). These findings suggest that the FLG-AS1/miR-23a-3p/HOXD10 axis possesses significant prognostic value in the context of surgical treatment for ESCC.

## Discussion

The lncRNA-miRNA-mRNA regulatory network is pivotal in the pathogenesis of cancer. Bioinformatics technologies serve as essential tools for uncovering potential molecular mechanisms underlying human diseases. In this study, we systematically explored lncRNAs and genes closely associated with ESCC by leveraging publicly available gene expression datasets from the GEO database. Subsequently, we employed the lncRNASNP2, Starbase, and TargetScan databases to predict the downstream miRNAs of lncRNAs and the upstream miRNAs of target genes. These molecular components were then integrated into a visual network using Cytoscape 3.7.1 software. Key pathways were identified by calculating node degree values, with the FLG-AS1/miR-23a-3p/HOXD10 pathway emerging as a prominent interaction within the top 10 nodes identified.

Gene regulation experiments demonstrated that FLG-AS1 functions as a competitive endogenous RNA, sequestering miR-23a-3p and consequently decreasing its availability, which in turn promoted the expression of HOXD10. Notably, both FLG-AS1 and HOXD10 exhibit anti-tumor properties in ESCC, while miR-23a-3p is implicated in carcinogenesis. Cisplatin, a cornerstone in cancer chemotherapy, has been associated with the development of drug resistance following prolonged usage. Our findings suggest that the overexpression of FLG-AS1 can counteract cisplatin resistance in ESCC cells by modulating the miR-23a-3p/HOXD10 axis. This insight offers a promising avenue for addressing chemotherapy resistance, enhancing the potential for improved therapeutic strategies in combating ESCC.

Altered energy metabolism represents one of the defining characteristics of cancer cells. In normal cellular physiology, energy is primarily derived from two pathways: anaerobic glycolysis and aerobic mitochondrial respiration. However, tumor cells preferentially utilize anaerobic glycolysis for energy production, even in the presence of oxygen. This metabolic reprogramming, known as the Warburg effect, allows them to convert glucose into pyruvate, ultimately producing lactic acid [18]. Such a mechanism enables tumor cells to sequester substantial amounts of glucose from their microenvironment to support their rapid proliferation, while simultaneously mitigating the production of deleterious reactive oxygen species typically generated via mitochondrial respiration. Furthermore, the substantial lactic acid output from anaerobic glycolysis contributes to the establishment of an acidic tumor microenvironment, which impairs T cell function and promotes enhanced growth and aggressiveness of cancer cells [19]. Consequently, targeting the energy metabolism of EC cells emerges as a pivotal strategy to thwart their immune evasion and curtail their growth and metastasis. In this context, the pivotal role of FLG-AS1 in inhibiting glucose uptake and lactic acid production in ESCC cells has been elucidated, acting through the modulation of the miR-23a-3p/HOXD10 axis. This finding provides a significant reference point for future ESCC therapeutic strategies.

Long non-coding RNAs (lncRNAs) have been extensively documented to play a pivotal role in the pathogenesis of EC. The upregulation of lncRNA MALR, stimulated by TNF-α, has been shown to activate the HIF-1α signaling pathway, thereby promoting the progression of EC [20]. Additionally, Wnt3a induces the alternative splicing of lncRNA-DGCR5, resulting in a shorter variant (DGCR5-S) that is associated with a poor prognosis in ESCC. The DGCR5-S variant inhibits the anti-inflammatory activity of tristetraprolin by safeguarding it from dephosphorylation by protein phosphatase 2 (PP2A), consequently fostering tumor-associated inflammation. Interrupting the splicing process of DGCR5 has been found to effectively hinder the growth of ESCC tumors [21]. In a retrospective study, five lncRNAs—PGM5-AS1, ADAMTS9-AS1, AP003548.1, LINC03016, and LINC01082—were identified as being downregulated in ESCC tissues in comparison to adjacent non-cancerous tissues. These lncRNAs demonstrated high accuracy in diagnosing ESCC and esophageal intraepithelial neoplasia [22]. Furthermore, FLG-AS1 appears to function as a tumor suppressor. Microarray analyses conducted by Lu et al. revealed that FLG-AS1 is differentially expressed between oral squamous cell carcinoma tissues and healthy oral mucosa [23]. Notably, FLG-AS1 is significantly downregulated in cervical cancer (CC) cells and tissues, exerting inhibitory effects on the proliferation of CC cells by negatively regulating the level of miR-147b. Additionally, CC patients with low expression of FLG-AS1 present with poorer prognoses [12], and diminished FLG-AS1 expression correlates significantly with shorter survival in patients with low-grade glioma [24]. However, the role of FLG-AS1 in the context of EC remains unreported.

MiR-23a-3p has been extensively characterized as a facilitator in various malignancies. Notably, it is significantly overexpressed in colon cancer (CLC), where its downregulation has been shown to mitigate the malignant traits of CLC cells by inhibiting the expression of NDRG4 [25]. Furthermore, miR-23a-3p is a recognized target of LINC00261; its overexpression can curtail the growth and metastasis of pancreatic cancer cells while promoting apoptotic pathways through the reduction of miR-23a-3p levels [26]. A study revealed that miR-23a-3p is upregulated in tumor tissues from high-grade serous ovarian cancer (HGSOC) patients who exhibit resistance to chemotherapy. This overexpression correlates significantly with diminished progression-free survival, thereby underscoring miR-23a-3p as a potential prognostic biomarker for HGSOC and a prospective target for mitigating platinum resistance [27]. Additionally, miR-23a-3p is found to be overexpressed in ESCC tumor tissues and cell lines compared to normal counterparts, a finding that correlates with enhanced tumor differentiation [28]. The depletion of miR-23a-3p has been documented to hinder the growth and spread of ESCC cells, accompanied by a reduction in glucose consumption and lactic acid production. Mechanistically, the lncRNA TMEM161B-AS1 elevates HIF1AN expression by competitively binding to miR-23a-3p, thereby obstructing the progression of ESCC [29]. While the role of miR-23a-3p in ESCC has been previously explored, our study seeks to elucidate a novel pathway through which it may contribute to ESCC pathology. The involvement of the FLG-AS1/miR-23a-3p/HOXD10 axis in ESCC remains unreported. It has been noted that fifteen members of the HOX gene family are dysregulated within ESCC cell lines, including HOXD10 [30]. Notably, HOXD10 is expressed at low levels in ESCC tissues and cell lines, while its overexpression has the potential to activate malignant phenotypes of ESCC cells through the inhibition of the PI3K/AKT/mTOR signaling pathway. This indicates that targeting HOXD10 may represent a viable therapeutic strategy for the treatment of ESCC [31].

Our retrospective study has revealed that ESCC patients exhibiting elevated levels of FLG-AS1 and HOXD10, in conjunction with diminished expression of miR-23a-3p, demonstrate a significantly improved 5-year survival rate post-surgery. This finding underscores the clinical significance of the FLG-AS1/miR-23a-3p/HOXD10 axis. In vitro experiments, FLG-AS1 upregulated HOXD10 expression by inhibiting miR-23a-3p, thereby suppressing the malignant functions of ESCC cells and reducing their cisplatin resistance. However, more in-depth in vivo experiments still need to be included in the future, which will better consolidate our results.

## Conclusion

Overall, the FLG-AS1/miR-23a-3p/HOXD10 pathway is implicated in the progression of ESCC, the development of cisplatin resistance, and patient prognosis. Thus, this study may pave the way for novel therapeutic strategies in the management of ESCC in the future.

## Electronic supplementary material

Below is the link to the electronic supplementary material.

Supplementary Material 1

Supplementary Material 2

## Data Availability

The datasets used and/or analysed during the current study are available from the corresponding author on reasonable request.

## References

[1] Wei MT, Friedland S. Early esophageal cancer: what the gastroenterologist needs to know. Gastroenterol Clin North Am. 2021;50(4):791–808. 10.1016/j.gtc.2021.07.004. 34717871 10.1016/j.gtc.2021.07.004

[2] Zhou N, Rajaram R, Hofstetter WL. Management of locally advanced esophageal Cancer. Surg Oncol Clin N Am. 2020;29(4):631–46. 10.1016/j.soc.2020.06.003. 32883463 10.1016/j.soc.2020.06.003

[3] Teng Y, Xia C, Cao M, Yang F, Yan X, He S, et al. Esophageal cancer global burden profiles, trends, and contributors. Cancer Biol Med. 2024;21(8):656–66. 10.20892/j.issn.2095-3941.2024.0145. 39066471 10.20892/j.issn.2095-3941.2024.0145 PMC11359494

[4] Weidenbaum C, Gibson MK. Approach to localized squamous cell Cancer of the esophagus. Curr Treat Options Oncol. 2022;23(10):1370–87. 10.1007/s11864-022-01003-w. 36042147 10.1007/s11864-022-01003-w PMC9526684

[5] Qu HT, Li Q, Hao L, Ni YJ, Luan WY, Yang Z, et al. Esophageal cancer screening, early detection and treatment: current insights and future directions. World J Gastrointest Oncol. 2024;16(4):1180–91. 10.4251/wjgo.v16.i4.1180. 38660654 10.4251/wjgo.v16.i4.1180 PMC11037049

[6] Qin S, Wang Y, Ma C, Lv Q. Competitive endogenous network of circrna, LncRNA, and MiRNA in osteosarcoma chemoresistance. Eur J Med Res. 2023;28(1):354. 10.1186/s40001-023-01309-x. 37717007 10.1186/s40001-023-01309-x PMC10504747

[7] Raziq K, Cai M, Dong K, Wang P, Afrifa J, Fu S. Competitive endogenous network of LncRNA, MiRNA, and mRNA in the chemoresistance of Gastrointestinal tract adenocarcinomas. Biomed Pharmacother. 2020;130:110570. 10.1016/j.biopha.2020.110570. 32763816 10.1016/j.biopha.2020.110570

[8] Wu X, Sui Z, Zhang H, Wang Y, Yu Z. Integrated analysis of lncRNA-Mediated CeRNA network in lung adenocarcinoma. Front Oncol. 2020;10:554759. 10.3389/fonc.2020.554759. 33042838 10.3389/fonc.2020.554759 PMC7523091

[9] Zhang XM, Wang J, Liu ZL, Liu H, Cheng YF, Wang T. LINC00657/miR-26a-5p/CKS2 CeRNA network promotes the growth of esophageal cancer cells via the MDM2/p53/Bcl2/Bax pathway. Biosci Rep. 2020;40(6). 10.1042/BSR20200525. 10.1042/BSR20200525 PMC7268253 32426838

[10] Lin C, Zhang S, Wang Y, Wang Y, Nice E, Guo C, et al. Functional role of a novel long noncoding RNA TTN-AS1 in esophageal squamous cell carcinoma progression and metastasis. Clin Cancer Res. 2018;24(2):486–98. 10.1158/1078-0432.CCR-17-1851. 29101304 10.1158/1078-0432.CCR-17-1851

[11] Yang F, Wen S, Zhang Y, Xu Y, Lv H, Zhu Y, et al. Identifying potential metastasis-related long non-coding RNAs, MicroRNAs, and message RNAs in the esophageal squamous cell carcinoma. J Cell Biochem. 2019;120(8):13202–15. 10.1002/jcb.28594. 30891809 10.1002/jcb.28594

[12] Gao M, Pang X, Ni S, Wei Z, Li D. Long Non-Coding RNA FLG-AS1 inhibits cervical Cancer progression through negatively modulating miR-147b. Ann Clin Lab Sci. 2024;54(2):149–55. 38802144

[13] Jin Z, Xu W, Yu K, Luo C, Luo X, Lian T, et al. The novel circFKBP8/miR-432-5p/E2F7 cascade functions as a regulatory network in breast cancer. Hereditas. 2024;161(1):27. 10.1186/s41065-024-00331-1. 39192374 10.1186/s41065-024-00331-1 PMC11348600

[14] Song Q, Liu H, Li C, Liang H. miR-33a-5p inhibits the progression of esophageal cancer through the DKK1-mediated Wnt/beta-catenin pathway. Aging. 2021;13(16):20481–94. 10.18632/aging.203430. 34426559 10.18632/aging.203430 PMC8436944

[15] Zhang N, Liu JF. MicroRNA (MiR)-301a-3p regulates the proliferation of esophageal squamous cells via targeting PTEN. Bioengineered. 2020;11(1):972–83. 10.1080/21655979.2020.1814658. 32970954 10.1080/21655979.2020.1814658 PMC8291791

[16] Tang L, Ruan Y, Wang B, Zhang M, Xue J, Wang T. Erianin inhibits the progression of DDP-resistant lung adenocarcinoma by regulating the Wnt/beta-catenin pathway and activating the caspase-3 for apoptosis in vitro and in vivo. Hereditas. 2024;161(1):48. 10.1186/s41065-024-00351-x. 39605083 10.1186/s41065-024-00351-x PMC11600767

[17] Wang J, Lai Z, Liu N, Wang Y, Li F, Song N, et al. A bioinformatics analysis of the target role of miRNA-431-5p on KLK6 in colorectal cancer. Hereditas. 2025;162(1):46. 10.1186/s41065-025-00395-7. 40156045 10.1186/s41065-025-00395-7 PMC11951700

[18] Chelakkot C, Chelakkot VS, Shin Y, Song K. Modulating Glycolysis to improve Cancer therapy. Int J Mol Sci. 2023;24(3). 10.3390/ijms24032606. 10.3390/ijms24032606 PMC9916680 36768924

[19] Paul S, Ghosh S, Kumar S. Tumor glycolysis, an essential sweet tooth of tumor cells. Semin Cancer Biol. 2022;86(Pt 3):1216–30. 10.1016/j.semcancer.2022.09.007. 36330953 10.1016/j.semcancer.2022.09.007

[20] Liu J, Liu ZX, Li JJ, Zeng ZL, Wang JH, Luo XJ, et al. The Macrophage-Associated LncRNA MALR facilitates ILF3 Liquid-Liquid phase separation to promote HIF1alpha signaling in esophageal Cancer. Cancer Res. 2023;83(9):1476–89. 10.1158/0008-5472.CAN-22-1922. 36264156 10.1158/0008-5472.CAN-22-1922

[21] Li Y, Chen B, Jiang X, Li Y, Wang X, Huang S, et al. A Wnt-induced lncRNA-DGCR5 splicing switch drives tumor-promoting inflammation in esophageal squamous cell carcinoma. Cell Rep. 2023;42(6):112542. 10.1016/j.celrep.2023.112542. 37210725 10.1016/j.celrep.2023.112542

[22] Zhou M, Bao S, Gong T, Wang Q, Sun J, Li J, et al. The transcriptional landscape and diagnostic potential of long non-coding RNAs in esophageal squamous cell carcinoma. Nat Commun. 2023;14(1):3799. 10.1038/s41467-023-39530-1. 37365153 10.1038/s41467-023-39530-1 PMC10293239

[23] Feng L, Houck JR, Lohavanichbutr P, Chen C. Transcriptome analysis reveals differentially expressed LncRNAs between oral squamous cell carcinoma and healthy oral mucosa. Oncotarget. 2017;8(19):31521–31. 10.18632/oncotarget.16358. 28415559 10.18632/oncotarget.16358 PMC5458226

[24] Nguyen QH, Nguyen T, Le DH. Identification and validation of a novel three hub long noncoding RNAs with m6A modification signature in Low-Grade gliomas. Front Mol Biosci. 2022;9:801931. 10.3389/fmolb.2022.801931. 35237657 10.3389/fmolb.2022.801931 PMC8882983

[25] Zuo H, Liu S, Li X, Hou G. miR-23a-3p promotes the development of colon cancer by inhibiting the expression of NDRG4. Clin Transl Oncol. 2023;25(4):933–40. 10.1007/s12094-022-02996-4. 36374403 10.1007/s12094-022-02996-4

[26] Wang X, Gao X, Tian J, Zhang R, Qiao Y, Hua X, et al. LINC00261 inhibits progression of pancreatic cancer by down-regulating miR-23a-3p. Arch Biochem Biophys. 2020;689:108469. 10.1016/j.abb.2020.108469. 32590069 10.1016/j.abb.2020.108469

[27] Todeschini P, Salviato E, Romani C, Raimondi V, Ciccarese F, Ferrari F, et al. Comprehensive profiling of Hypoxia-Related MiRNAs identifies miR-23a-3p overexpression as a marker of platinum resistance and poor prognosis in High-Grade serous ovarian Cancer. Cancers (Basel). 2021;13(13). 10.3390/cancers13133358. 10.3390/cancers13133358 PMC8268862 34283087

[28] Zhu L, Jin L, Jiang R, Wang Q, Jiang J, Mao C, et al. [Correlations between MiRNAs and TGF-beta1 in tumor microenvironment of esophageal squamous cell cancer]. Xi Bao Yu Fen Zi Mian Yi Xue Za Zhi. 2013;29(5):524–8. 23643275

[29] Cell Mol CJ. Med. 2023;27(4):591–2. 10.1111/jcmm.17661 10.1111/jcmm.17661 PMC9930414 36790069

[30] Gu ZD, Chen XM, Zhang W, Gu J, Chen KN. [Expression of 39 HOX genes in esophageal cancer cell lines]. Zhonghua Wei Chang Wai Ke Za Zhi. 2007;10(4):365–7. 17659465

[31] Zhang J, Liu S, Zhang D, Ma Z, Sun L. Homeobox D10, a tumor suppressor, inhibits the proliferation and migration of esophageal squamous cell carcinoma. J Cell Biochem. 2019;120(8):13717–25. 10.1002/jcb.28644. 30938888 10.1002/jcb.28644

