# Supplementary figures and images for: LncRNA FLG-AS1 inhibits esophageal squamous cell carcinoma by regulating the miR-23a-3p/HOXD10 axis
Source: Hereditas. 2025 Jun 3;162:96. doi: 10.1186/s41065-025-00461-0 (PMC12131396; doi:10.1186/s41065-025-00461-0)

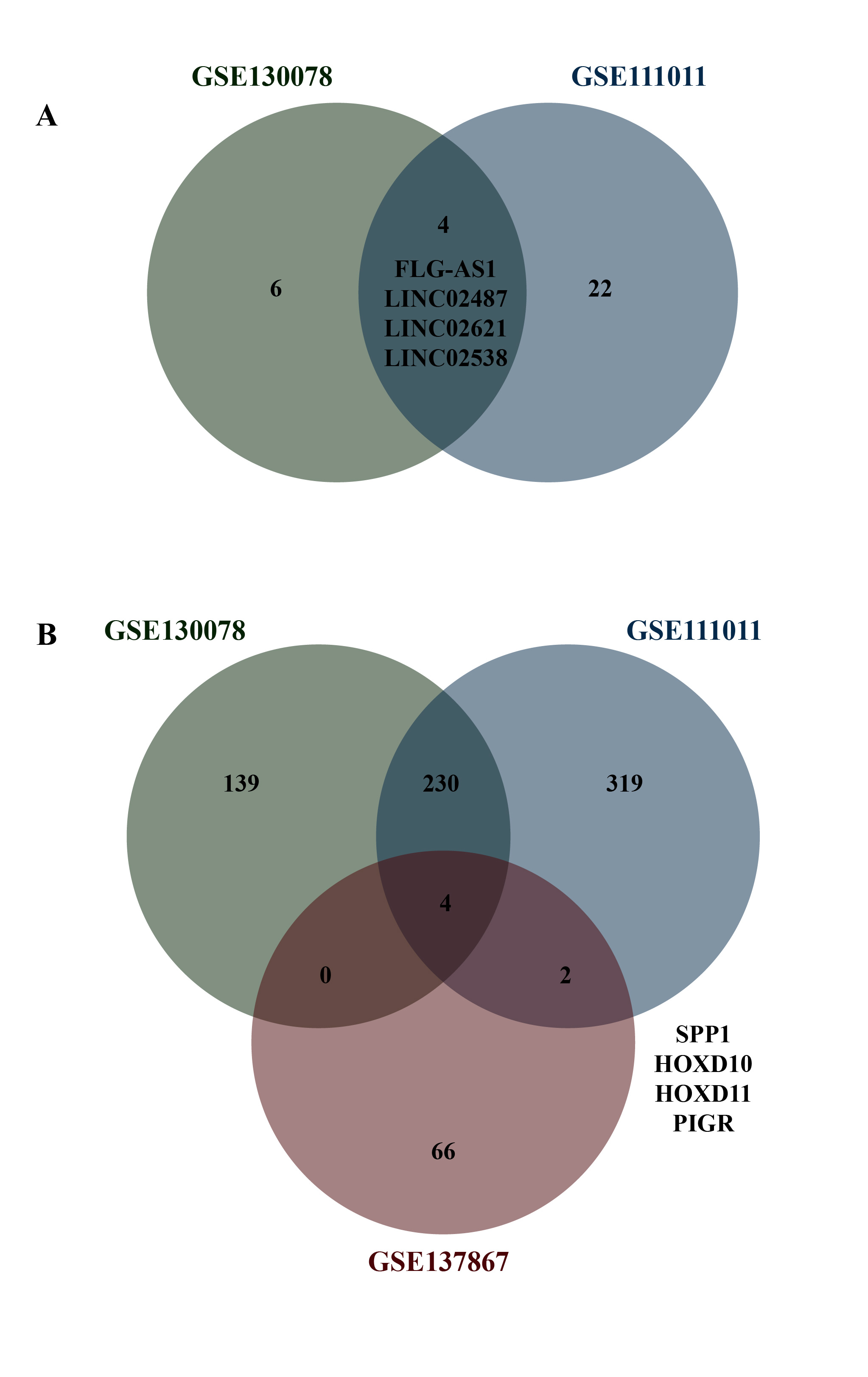

Supplement: Supplementary file 1 — Supplementary Material 1 [file 41065_2025_461_MOESM1_ESM.jpg]
