# Supplementary material for: LncRNA FLG-AS1 inhibits esophageal squamous cell carcinoma by regulating the miR-23a-3p/HOXD10 axis
Source: Hereditas. 2025 Jun 3;162:96. doi: 10.1186/s41065-025-00461-0 (PMC12131396; doi:10.1186/s41065-025-00461-0)
Supplement: Supplementary file 2 — Supplementary Material 2 [file 41065_2025_461_MOESM2_ESM.docx]

**Supplementary Table 1.** The primer sequences

| Gene Name | Forward Primer | Reverse Primer |
| --- | --- | --- |
| FLG-AS1 | 5'-GGTCTCACAAAGAGGATACCTG-3' | 5'-TGAACCTGGACAAGTCACTAAAG-3' |
| miR-23a-3p | 5'-CCAGGAACCCCTCCTTACTC-3' | 5'-TCTAGGGATGGTCCGAAGGA-3' |
| HOXD10 | 5'- GACATGGGGACCTATGGAATGC -3' | 5'- CGGATCTGTCCAACTGTCTACT -3' |
| U6 | 5'-CTCGCTTCGGCAGCACA-3' | 5'-AACGCTTCACGAATTTGCGT-3' |
| GAPDH | 5'-GTAACCCGTTGAACCCCATT-3' | 5'-CCATCCAATCGGTAGTAGCG-3' |
| GLUT3 | 5’-AACACTGGGGTCATCAATGCTC-3’ | 5’-AGAGACGTGAGCAGCACCTCA-3’ |
| MDR1 | 5'-ATAATGCGACAGGAGATAGG-3' | 5'-CCAAAATCACAAGGGTTAGC-3' |
